# Blocking of Tim‐3 Ameliorates Spinal Cord Ischemia‐Reperfusion Injury Through Inhibiting Neuroinflammation and Promoting M1‐to‐M2 Phenotypic Polarization of Microglia

**DOI:** 10.1002/iid3.70084

**Published:** 2024-12-06

**Authors:** Zhenxing Li, Binbin Zhou, Guanghui Chen, Xiangyu Yang, Han Su, Bolin Li

**Affiliations:** ^1^ Department of Rehabilitation First Affiliated Hospital of the Guangxi University of Chinese Medicine Nanning Guangxi China; ^2^ Department of Tuina Ruikang Hospital Affiliated to Guangxi University of Chinese Medicine Nanning Guangxi China; ^3^ Department of Emergency First Affiliated Hospital of the Guangxi University of Chinese Medicine Nanning Guangxi China

## Abstract

**Background:**

Blocking of Tim‐3 exerts therapeutic effects in a series of ischemia‐reperfusion injury (IRI).

**Methods:**

In this work, a cross‐clamped aortic arch was conducted to establish SCIRI rat model. Besides, rat spinal microglia was subjected to OGD/R to mimic I/R‐like conditions in vitro. The in vivo and in vitro therapeutic effects of Tim‐3 antibody in SCIRI were investigated from these aspects: neuronal apoptosis, neuroinflammation, microglia activation, and polarization.

**Results:**

It was verified that Tim‐3 was highly expressed in spinal cord tissues of SCIRI rats and blocking of Tim‐3 attenuated SCIRI‐induced pathological injury, neuronal apoptosis, neuroinflammation, and microglia activation (M1 polarization). In addition, it was verified that Tim‐3 was highly expressed in OGD/R‐treated rat spinal microglia and blocking of Tim‐3 attenuated OGD/R‐induced inflammation and spinal microglia activation (M1 polarization).

**Conclusions:**

Tim‐3 antibody can exert therapeutic effects in SCIRI through inhibiting neuroinflammation and promoting microglia polarization from M1 to M2 phenotype.

## 1 Introduction

As a destructive complication of aortic aneurysm repair or spinal cord decompression surgeries, spinal cord ischemia‐reperfusion injury (SCIRI) can lead to irreversible death of spinal cord neurons, spinal cord dysfunction, and even fatal paralysis due to the extreme sensitivity of spinal cord neurons to ischemia and hypoxia [1, 2]. A diverse of pathological processes including apoptosis, inflammatory reactions, oxidative stress, and microglia activation have close correlations with SCIRI [3, 4]. Till now, there is a lack of effective strategies to ameliorate SCIRI and exploration of SCIRI therapies remains one hotspot in basic and clinical research.

T cell immunoglobulin and mucin domain 3 (Tim‐3), a member of Tim family, is initially identified as expressed on activated T helper type 1 (Th1) cells [5]. Despite current research concerning Tim‐3 focus on its role in Th1 cells, Tim‐3 has been currently discovered to be expressed on and to exert diversified functions in other types of cells [6, 7]. Evidence gathered suggests that Tim exerts double roles in regulating inflammation [8]. Tim‐3 expressed on Th1 cells can inhibit the inflammatory response by eliminating Th1 cells [9, 10] while Tim‐3 expressed on macrophages, microglia, and dendritic cells can promote inflammatory response [11]. Besides, Tim‐3 has either a negative or positive regulatory function on diverse cells during innate and adaptive immune responses [12, 13]. Interestingly, it has been reported that ischemic postconditioning may reduce infarction by inactivating galectin‐9/Tim‐3 signaling pathway as well as attenuating the inflammatory factors iNOS [14]. miR‐330‐5p can ameliorate NLRP3 inflammasome‐mediated myocardial ischaemia‐reperfusion injury (IRI) by suppressing Tim‐3 [15]. Nateglinide can exert neuroprotective effects in rat's hippocampus suffered from focal cerebral IRI by inactivating HIF‐1α/Tim‐3 inflammatory pathway [16]. Blockade of Tim‐3 can reduce infarct size, neuronal cell death, oedema formation, and neutrophil infiltration in cerebral hypoxia‐ischaemia mice [17]. Tim‐3 can exacerbate kidney IRI through activation of TLR‐4/NF‐κB signaling pathway and NLR‐C4 inflammasome [18]. Nevertheless, the functional role of Tim‐3 and the intrinsic mechanisms underlying the protective effects of Tim‐3 deficiency in SCIRI have not been fully elucidated till now.

In the present research, SCIRI rat model and OGD/R‐treated rat spinal microglia were employed to investigate the in vivo and in vitro therapeutic effects of Tim‐3 antibody in SCIRI from these aspects: neuronal apoptosis, neuroinflammation, microglia activation, and polarization.

## 2 Materials and Methods

### 2.1 Clinical Specimen Collection

SCI patients admitted to First Affiliated Hospital of the Guangxi University of Chinese Medicine were screened. 16 patients who met the inclusion criteria but not the exclusion criteria were included in the study and 6 volunteers were recruited to join the study as controls. Blood samples were immediately frozen and stored at −80°C for further study. The study was approved and authorized by Ethic Committee of First Affiliated Hospital of the Guangxi University of Chinese Medicine, and patients or family members gave full informed and written consent to the study.

### 2.2 Animals

SD rats (200–250 g) were housed in a standard environmental condition for 1 week before surgical operation. Animal experiments were approved by the Ethics Committee of First Affiliated Hospital of the Guangxi University of Chinese Medicine.

### 2.3 Establishment of SCIRI Rat Model [19]

A cross‐clamped aortic arch was conducted to induce SCIRI in rats. Upon anesthesia by intraperitoneal injection with 4% sodium pentobarbital (50 mg/kg), rats received laparotomy. Briefly, the aortic arch was exposed through a cervicothoracic incision and then cross‐clamped for 14 min between the left carotid artery and the left subclavian artery to induce ischemia. After ischemia confirmation [90% reduction of the flow assessed by a laser Dopplerblood flow monitor, clamp removal was performed, followed by 6‐ or 12‐h reperfusion. Rats were killed through subjecting to a lethal dose of pentobarbital and spinal cord (L4–L6) specimens were sampled for subsequent examination.

### 2.4 Experimental Design in Vivo

Groups were enrolled: (I) Control group (*n* = 6); (II) I/R group (*n* = 6), SCIRI rat model receiving no treatment; (III) I/R + IgG anti‐body group (*n* = 6), SCIRI rat model receiving intrathecal injection with 20 μg/10 μL of control IgG anti‐body; (IV) I/R + Tim‐3 anti‐body group (*n* = 6), SCIRI rat model receiving intrathecal injection with 20 μg/10 μL of Tim‐3 anti‐body. Intrathecal infusions were administered for 5 consecutive days before surgery [20].

### 2.5 Oxygen and Glucose Deprivation/Reoxygenation (OGD/R) Treatment [21]

To mimic I/R‐like conditions in vitro, rat spinal microglia was subjected to OGD/R. First, rat spinal microglia were maintained in glucose‐free DMEM with 94% N_2_, 5% CO_2_, and 1% O_2_ at 37°C for 6 h. Afterwards, the culture medium was replaced with glucose‐containing DMEM, and cells were cultured with 5% CO_2_, 95% O_2_ at 37°C for 24 h.

### 2.6 Experimental Design in Vitro

Groups were enrolled: (I) Control group; (II) OGD/R group; (III) OGD/R + IgG anti‐body group; (IV) OGD/R + Tim‐3 anti‐body group.

### 2.7 Hematoxylin and Eosin (H&E) Staining

Spinal cord tissues were fixed by 4% paraformaldehyde, embedded in paraffin, sectioned, dewaxed with dimethyl benzene, and then dehydrated with graded ethanol. Subsequently, H&E staining was performed and the stained sections were photographed under an optical microscope to observe the morphological appearance of neurons.

### 2.8 TdT‐Mediated dUTP Nick‐End Labeling (TUNEL) Staining

Apoptosis of spinal cord tissues was measured using TUNEL Apoptosis Detection kit. In brief, paraffin‐embedded spinal cord tissue slices were dewaxed with dimethyl benzene, dehydrated with graded ethanol, then labeled with TUNEL in the dark at 37°C for 1 h and counterstained with DAPI staining solution in the dark at room temperature for 5 min. Samples were observed and photographed under a fluorescence microscope.

### 2.9 Enzyme‐Linked Immunosorbent Assay (ELISA)

The contents of TNF‐α, IL‐1β, and IL‐6 in rat serum were determined using ELISA kits in line with the manufacturer's instructions.

### 2.10 Immunohistochemistry

Paraffin‐embedded spinal cord tissue slices were dewaxed with dimethyl benzene, dehydrated with graded ethanol, soaked in 3% H_2_O_2_ for 20 min and then reacted with antigen repair buffer at boiling for 10 min. After incubation with normal goat serum blocking solution, the tissue slices were incubated with primary antibody against IBA‐1 at 4°C overnight, washed with PBS, further incubated with Horseradish Peroxidase (HRP) conjugated secondary goat anti‐rabbit immunoglobulin G (IgG) antibody at room temperature for 1 h and counterstained with hematoxylin at room temperature for 4 min. Samples were mounted with 10% glycerinum/PBS and observed under an optical microscope.

### 2.11 Immunofluorescence

Tissue slices or rat spinal microglia on cover glass were permeabilized by 0.5% Triton X‐100. After incubation with normal goat serum blocking solution, slices or cells were incubated with primary antibodies against IBA‐1, iNOS, Arg‐1 at 4°C overnight, further incubated with fluorescein isothiocyanate (FITC) conjugated secondary antibody at room temperature for 1 h and counterstained with DAPI at room temperature for 5 min. Images were observed and photographed under a fluorescence microscope.

### 2.12 Reverse‐Transcription Quantitative Polymerase Chain Reaction (RT‐qPCR)

Total RNA extracted from tissues or cells using TRIzol reagent was reversely transcribed to cDNA using PrimeScript™ RT Master Mix and PCR amplification was carried out using TB Green® Premix Ex Taq™ II on the Applied Biosystems 7300 Real‐Time PCR System. Glyceraldehyde 3‐phosphate dehydrogenase (GAPDH) served as the internal reference and the relative mRNA expression levels of Tim‐3 and IBA‐1 were assessed by $2^{‐∆∆C_{t}}$ method [22].

### 2.13 Western Blot Assay

Protein concentration of total protein extracted from tissues or cells using RIPA lysis buffer was determined by BCA method. Next, equal amount of protein samples were separated by SDS‐PAGE and transferred onto PVDF membranes. After blocking with 5% BSA at room temperature for 1 h, membranes were incubated with primary antibodies against Tim‐3, TNF‐α, IL‐1β, IL‐6, p‐p65, p65, IBA‐1, iNOS, CD86, Arg‐1, CD206, Bcl‐2, Bax, cleaved caspase‐3, caspase‐3, and GAPDH at 4°C overnight and further incubated with HRP conjugated secondary goat anti‐rabbit IgG antibody at room temperature for 1 h. Protein signals were developed using an enhanced ECL kit and protein band intensities were analyzed using Image J.

### 2.14 Statistical Analysis

Data were analyzed using SPSS 23.0 software and presented as mean value ± SD. Comparisons among multiple groups were conducted using one‐way analysis of variance followed by Tukey's post hoc test and comparisons between two groups were conducted using unpaired Student's *t*‐test. Significant difference was set at *p* < 0.05.

## 3 Results

### 3.1 Tim‐3 Is Highly Expressed in SCI Patients and Spinal Cord Tissues of SCIRI Rats

**Figure 1:**
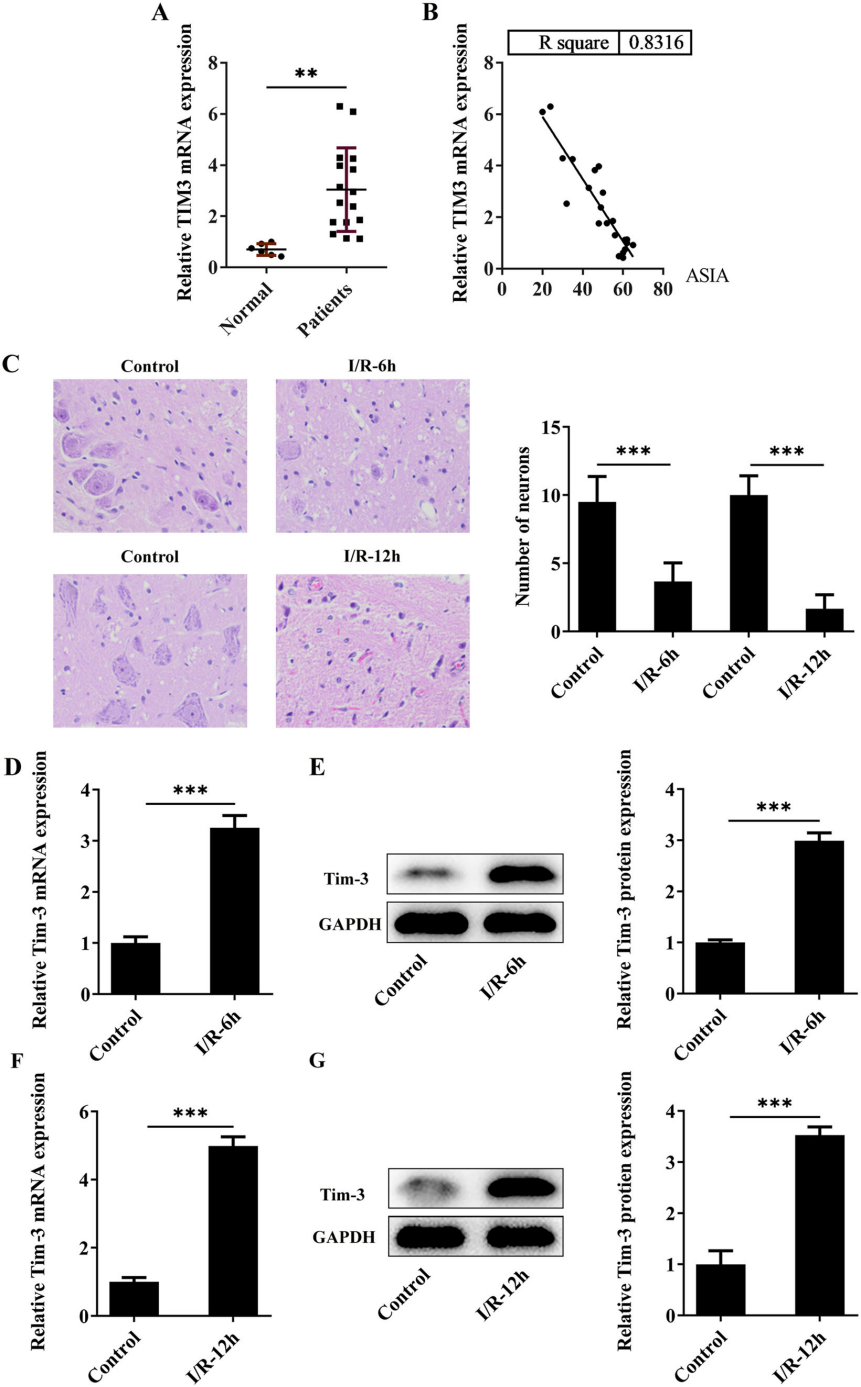
Tim‐3 is highly expressed in spinal cord tissues of SCIRI rats. A cross‐clamped aortic arch was conducted to induce SCIRI in rats. Spinal cord specimens were sampled at 6 or 12 h post‐SCIRI. (A) Serum Tim‐3 in SCI patients and normal volunteers was detected by RT‐qPCR. (B) Correlation between serum Tim‐3 and ASIA score. (C) The number of intact neurons in the anterior horn of spinal cord was detected by H&E staining. (D–G) Tim‐3 mRNA and protein levels in spinal cord tissues were detected by RT‐qPCR and western blot assay, respectively. **p < 0.01, ***p < 0.001.

Blood samples of SCI patients and normal volunteers were collected and it was verified that Tim‐3 had a higher expression level in SCI patients in comparison with that in the controls (Figure 1A). The correlation between serum Tim‐3 and ASIA score was analyzed and results indicated that ASIA score showed a downward trend with the increase of serum Tim‐3 levels (Figure 1B). The injury degree was positively correlated with serum Tim‐3 expression levels. A cross‐clamped aortic arch was conducted to induce SCIRI in rats. Spinal cord specimens were sampled at 6 or 12 h after reperfusion for subsequent examination. H&E staining showed a reduction in the number of intact neurons in the anterior horn of spinal cord after SCIRI, especially at 12 h post‐SCIRI (Figure 1C). Besides, in comparison with control rats, higher expression levels of Tim‐3 were observed in spinal cord tissues of SCIRI rats, especially in spinal cord tissues of SCIRI rats subjected to 12‐h reperfusion (Figure 1D–G). Hence, SCIRI rats subjected to 12‐h reperfusion was selected for the following study.

### 3.2 Tim‐3 Inhibition Mitigates Pathological Injury After SCIRI

**Figure 2:**
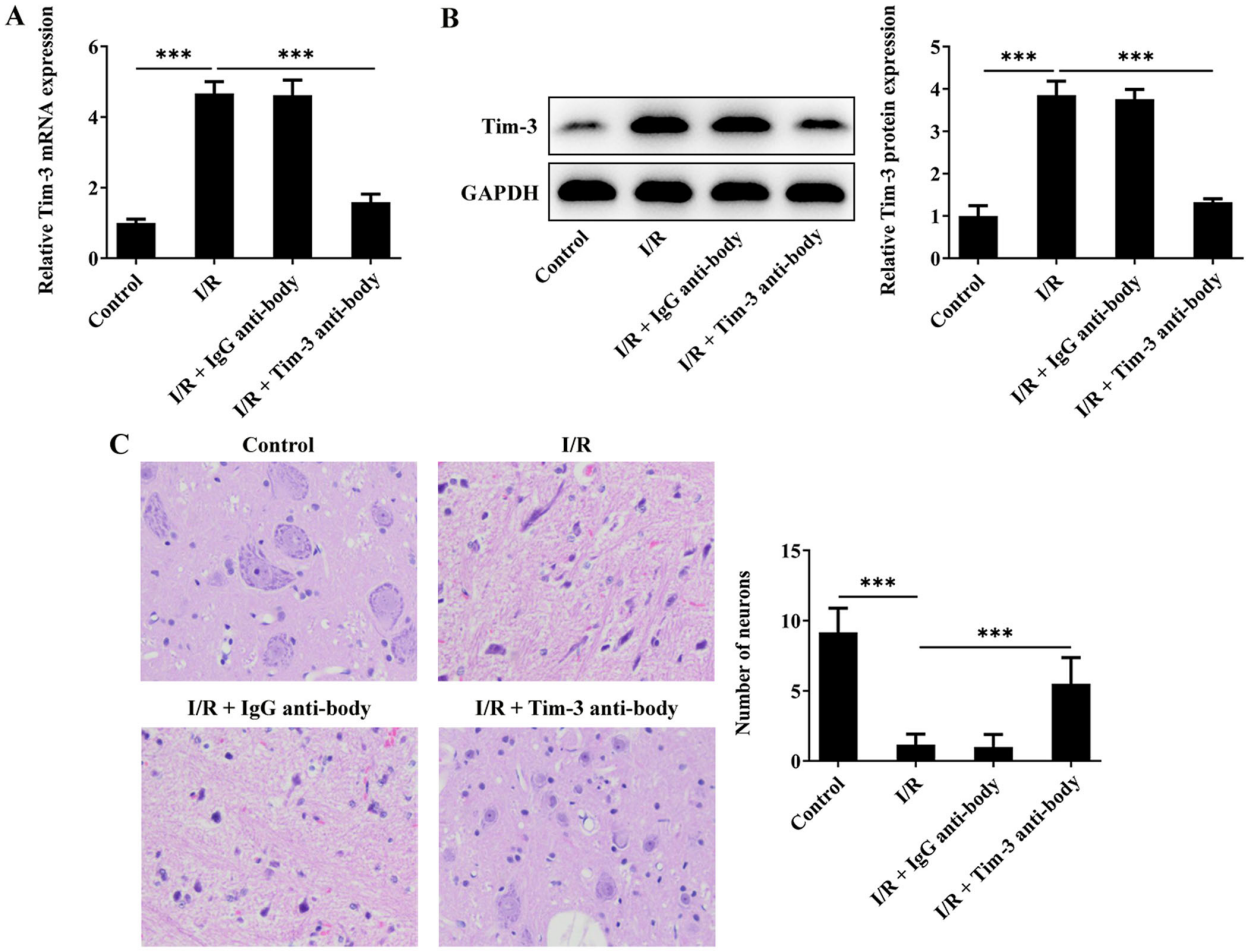
Tim‐3 inhibition mitigates pathological injury of SCIRI rats. SCIRI rats were intrathecally injected with Tim‐3 antibody or lgG antibody. (A, B) Tim‐3 mRNA and protein levels in spinal cord tissues were detected by RT‐qPCR and western blot assay, respectively. (C) The number of intact neurons in the anterior horn of spinal cord was detected by H&E staining. ***p < 0.001.

To thoroughly investigate the biological role of Tim‐3 in SCIRI, SCIRI rats were intrathecally injected with Tim‐3 antibody to block Tim‐3 expression (Figure 2A,B). Blocking of Tim‐3 obviously elevated the number of neurons with normal morphology in the anterior horn of spinal cord in comparison with SCIRI rats (Figure 2C).

### 3.3 Tim‐3 Inhibition Ameliorates Neuronal Apoptosis After SCIRI

**Figure 3:**
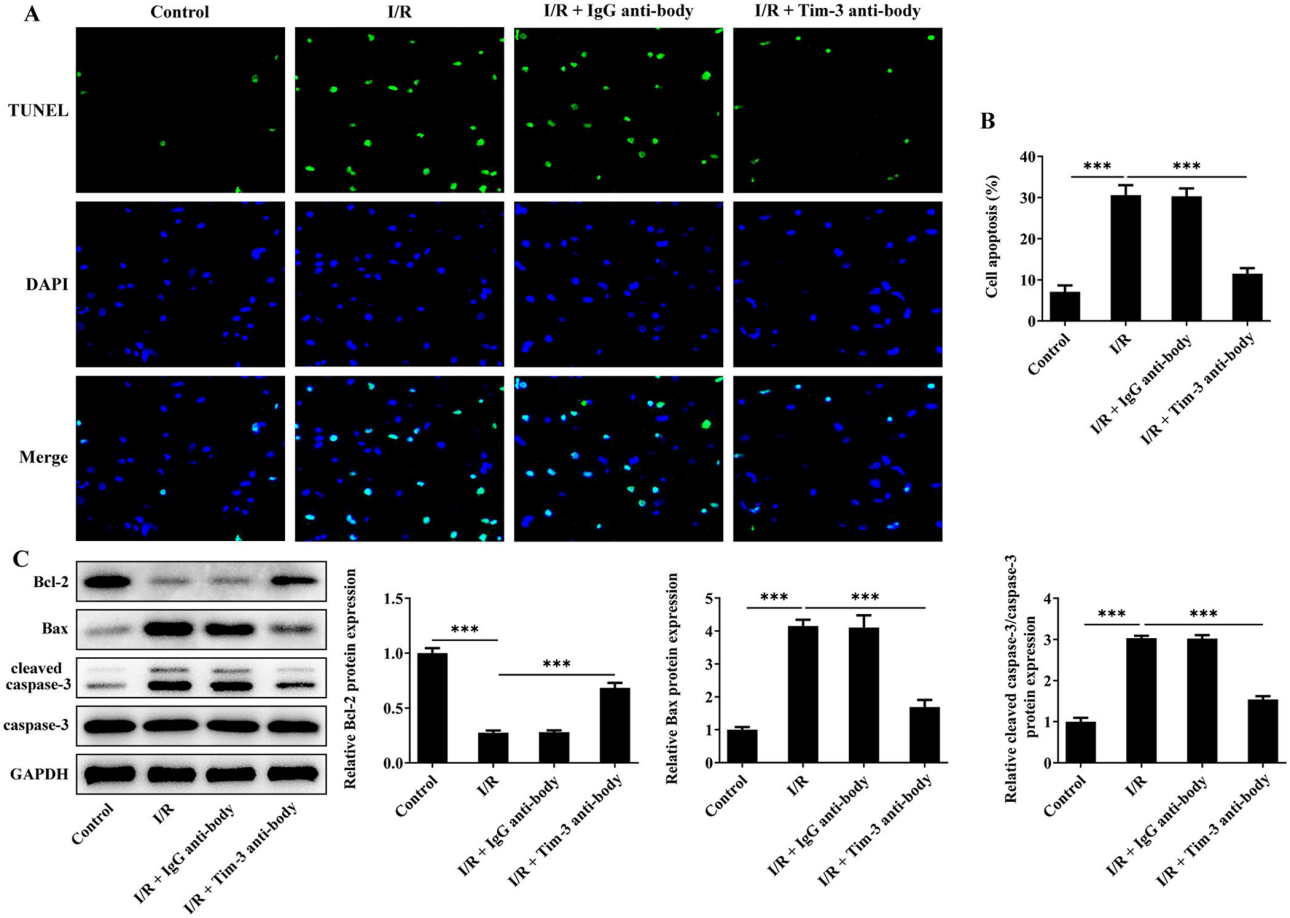
Tim‐3 inhibition ameliorates neuronal apoptosis after SCIRI. SCIRI rats were intrathecally injected with Tim‐3 antibody or lgG antibody. (A, B) Neuronal apoptosis in spinal cord tissues was detected by TUNEL staining. (C) Expressions of Bcl‐2, Bax, cleaved caspase‐3, and caspase‐3 in spinal cord tissues were detected by western blot assay. ***p < 0.001.

Neuronal apoptosis in spinal cord tissues was represented by TUNEL‐positive signals. Results showed that the apoptotic rate was markedly increased in SCIRI rats whereas blocking of Tim‐3 partially restored the increased apoptotic rate after SCIRI (Figure 3A,B). Similarly, Tim‐3 inhibition increased expression of Bcl‐2 and decreased expressions of Bax and cleaved caspase‐3, partially abolishing SCIRI‐induced neuronal apoptosis (Figure 3C).

### 3.4 Tim‐3 Inhibition Alleviates Neuroinflammation After SCIRI

**Figure 4:**
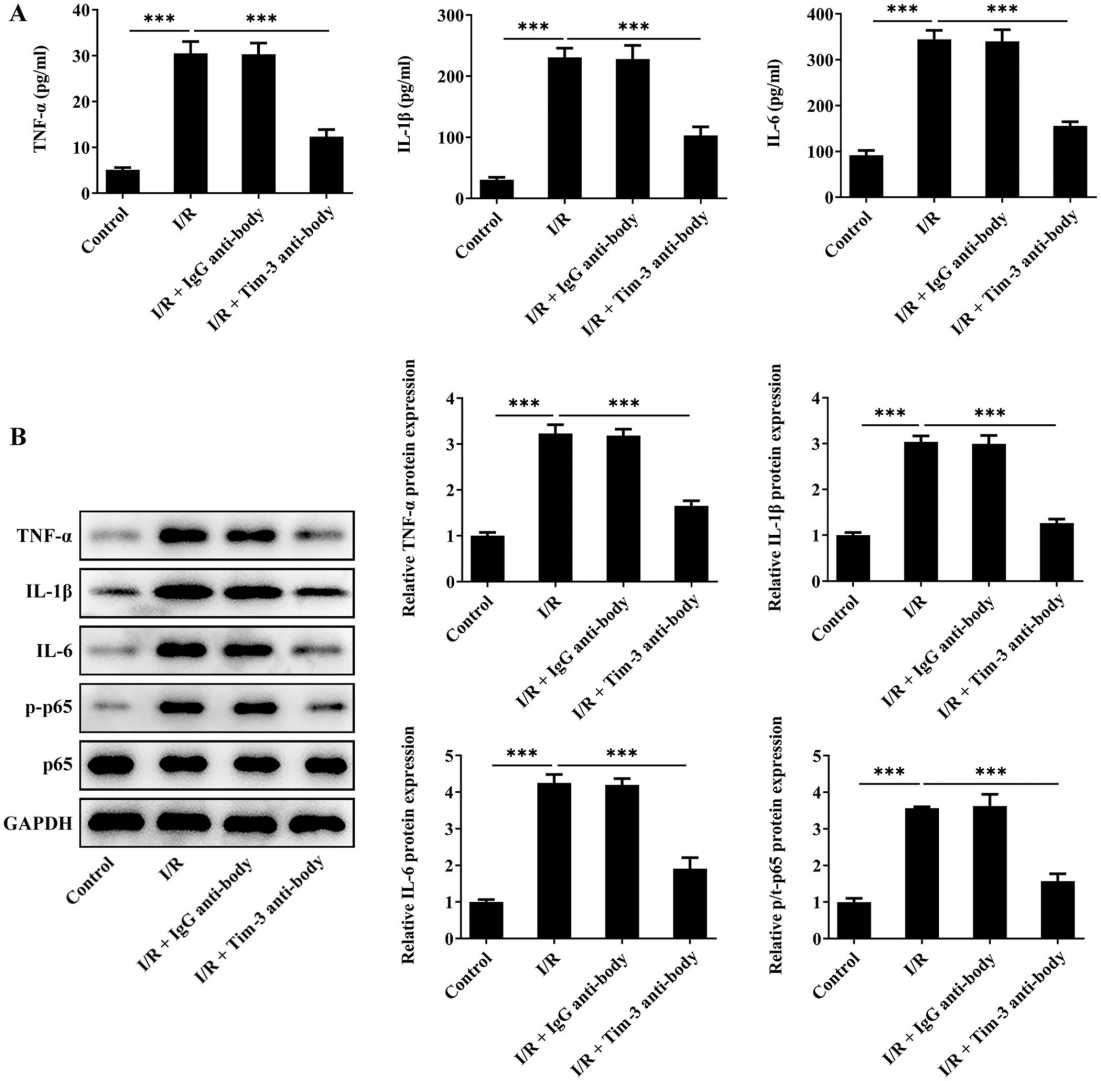
Tim‐3 inhibition alleviates neuroinflammation after SCIRI. SCIRI rats were intrathecally injected with Tim‐3 antibody or lgG antibody. (A)The contents of TNF‐α, IL‐1β, and IL‐6 in rat serum were detected using ELISA kits. (A, B) Expressions of TNF‐α, IL‐1β, IL‐6, p‐p6,5 and p65 in spinal cord tissues were detected by western blot assay. ***p < 0.001.

Obvious neuroinflammation was observed in rats after SCIRI. Blocking of Tim‐3 reduced the levels of TNF‐α, IL‐1β, and IL‐6 in SCIRI rat serum and inhibited the expressions of TNF‐α, IL‐1β, IL‐6, and p‐p65 in spinal cord tissues of SCIRI rats, partially abolishing SCIRI‐induced neuroinflammation (Figure 4A,B).

### 3.5 Tim‐3 Inhibition Attenuates Microglia Activation (M1 Polarization) After SCIRI

**Figure 5:**
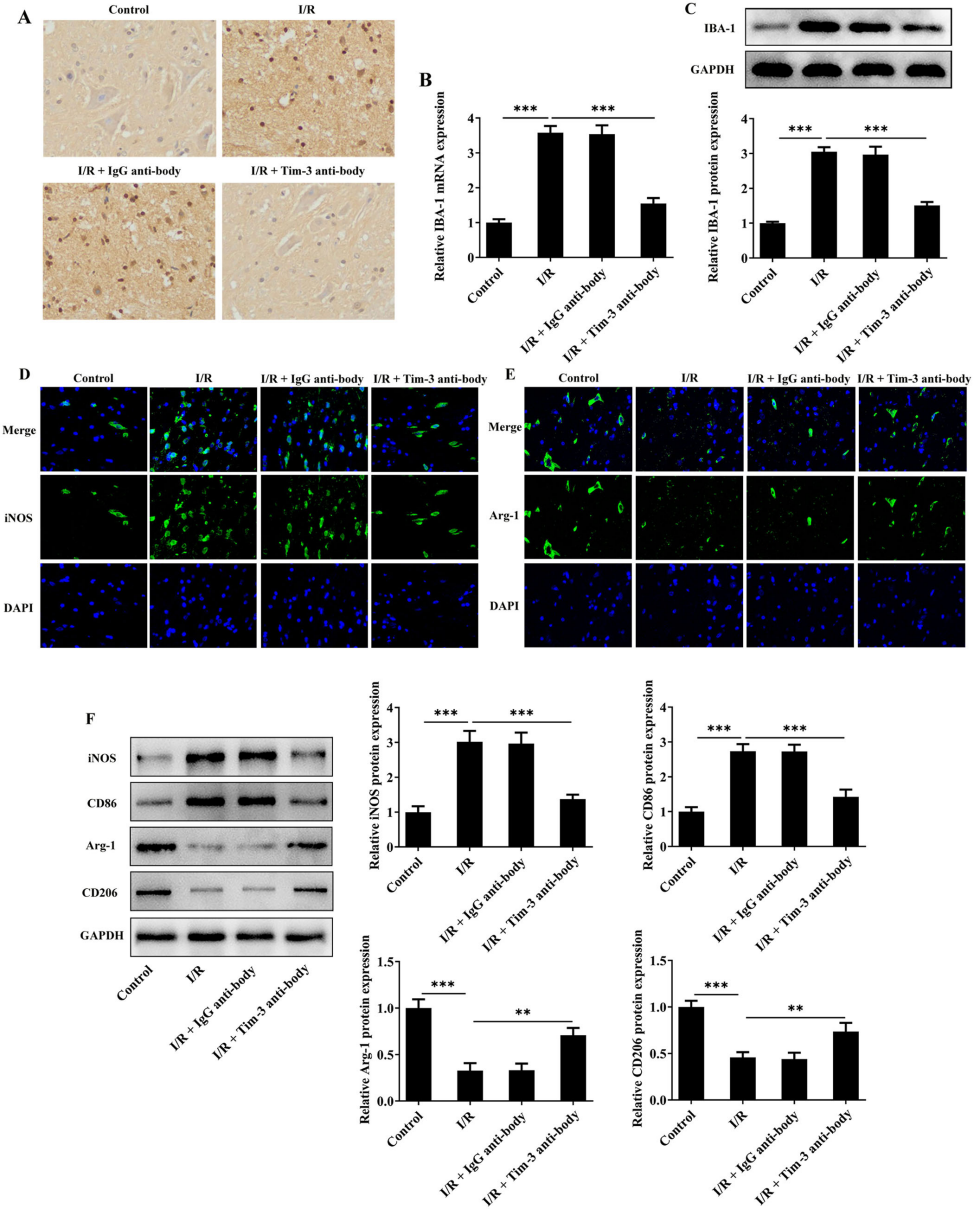
Tim‐3 inhibition attenuates microglia activation (M1 polarization) after SCIRI. SCIRI rats were intrathecally injected with Tim‐3 antibody or lgG antibody. (A) IBA‐1 expression in spinal cord tissues was detected by immunohistochemistry staining. (B, C) IBA‐1 mRNA and protein levels in spinal cord tissues were detected by RT‐qPCR and western blot assay, respectively. (D) iNOS expression in spinal cord tissues was detected by immunofluorescence staining. (E) Arg‐1 expression in spinal cord tissues was detected by immunofluorescence staining. (F) Expressions of iNOS, CD86, Arg‐1, CD206 in spinal cord tissues were detected by western blot assay. **p < 0.01, ***p < 0.001.

By immunohistochemistry staining, RT‐qPCR, and western blot assay, dramatically elevated expression of IBA‐1, a marker of activated microglia, was observed in spinal cord tissues of SCIRI rats. Blocking of Tim‐3 partially restored the increased activation of microglia, demonstrated by reduced IBA‐1 expression (Figure 5A–C). By immunofluorescence staining and western blot assay, markers characteristic of M1 microglia (iNOS, CD86) showed a marked elevation in spinal cord tissues of SCIRI rats, and indicators for M2 microglia (Arg‐1, CD206) were notably reduced in spinal cord tissues of SCIRI rats, indicating that SCIRI steered microglia polarization towards the phenotype of M1. Furthermore, blocking of Tim‐3 promoted microglia polarization from M1 to M2 phenotype, demonstrated by reduced expressions of iNOS and CD86 and elevated expressions of Arg‐1 and CD206 (Figure 5D–F).

### 3.6 Tim‐3 Is Highly Expressed in OGD/R‐Treated Rat Spinal Microglia

**Figure 6:**
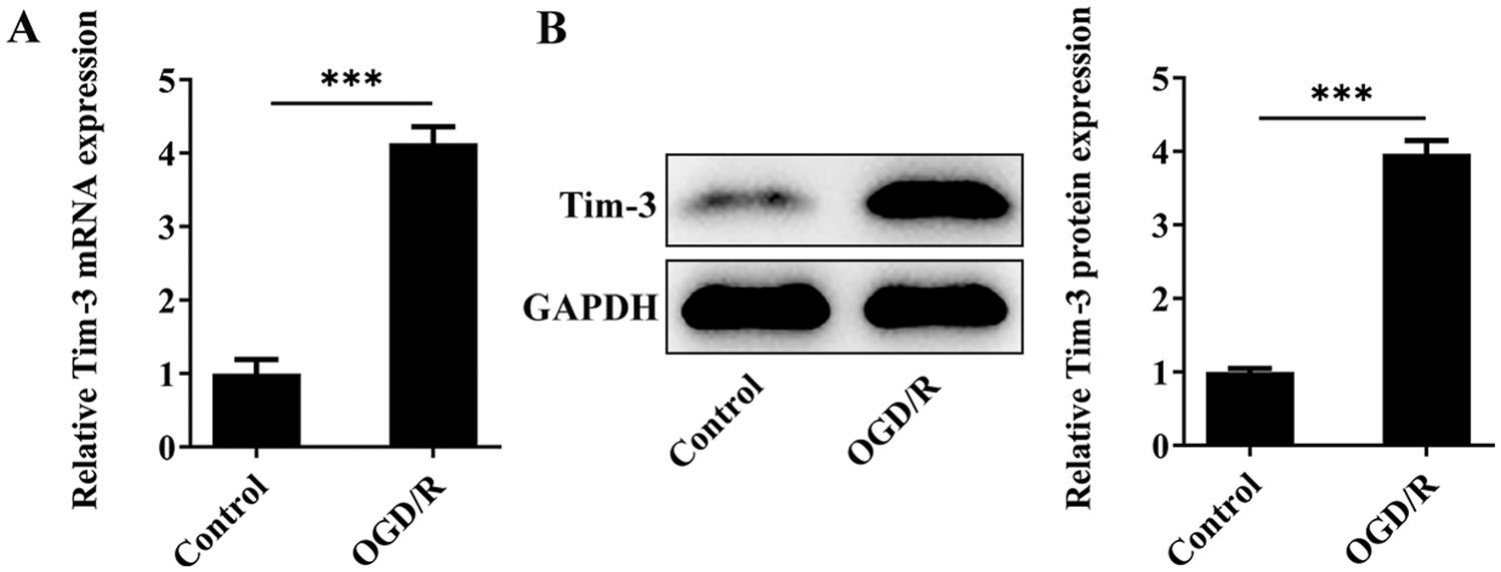
Tim‐3 is highly expressed in OGD/R‐treated rat spinal microglia. Rat spinal microglia was subjected to OGD/R to mimic I/R‐like conditions in vitro. (A, B) Tim‐3 mRNA and protein levels in rat spinal microglia were detected by RT‐qPCR and western blot assay, respectively. ***p < 0.001.

To mimic I/R‐like conditions in vitro, rat spinal microglia was subjected to OGD/R. In comparison with control, OGD/R treatment caused a higher expression level of Tim‐3 in rat spinal microglia (Figure 6A,B).

### 3.7 Tim‐3 Inhibition Alleviates Inflammation in OGD/R‐Treated Rat Spinal Microglia

**Figure 7:**
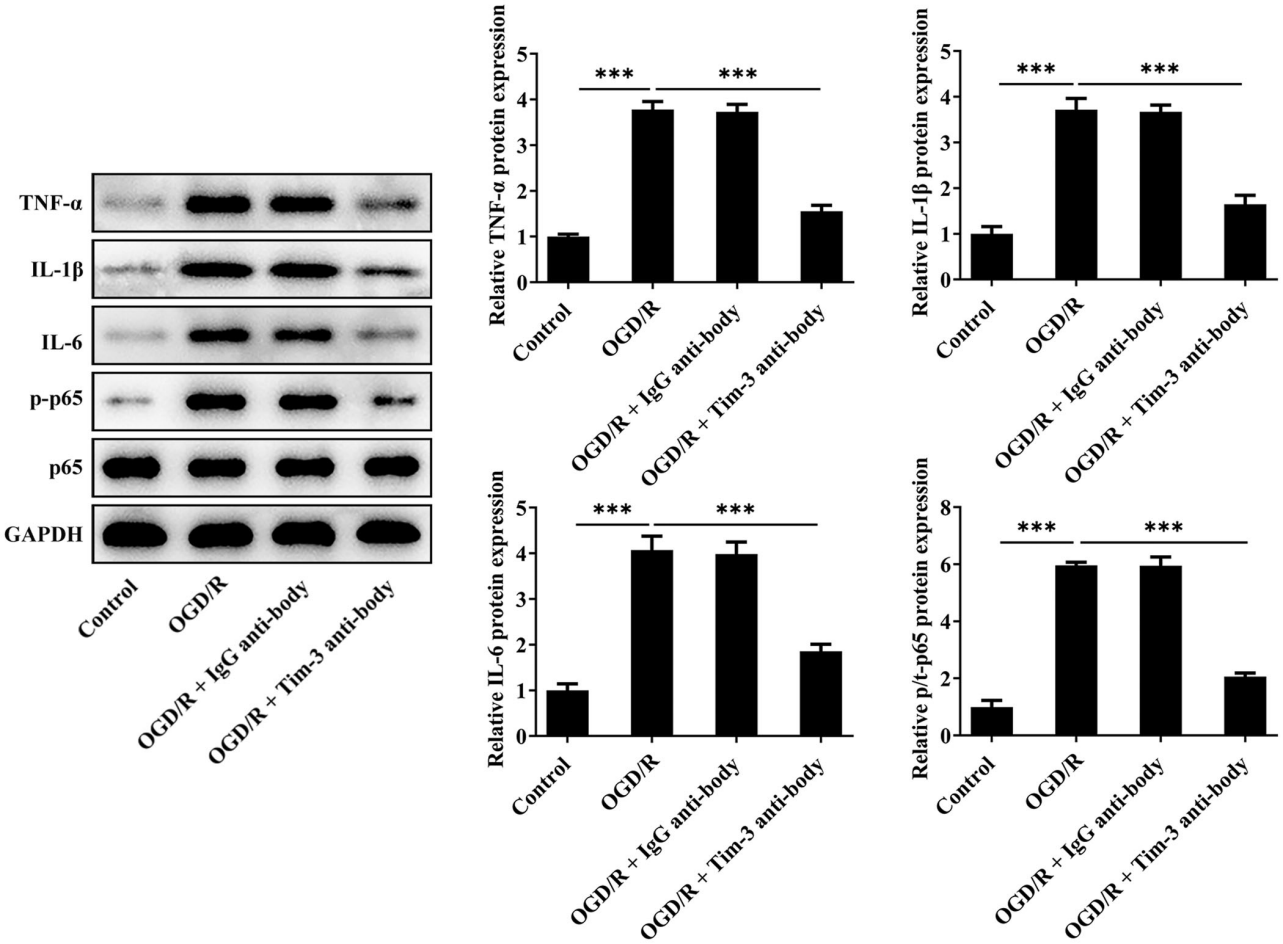
Tim‐3 inhibition alleviates inflammation in OGD/R‐treated rat spinal microglia. OGD/R‐treated rat spinal microglia was administrated with Tim‐3 antibody or lgG antibody. Expressions of TNF‐α, IL‐1β, IL‐6, p‐p65, and p65 in rat spinal microglia were detected by western blot assay. ***p < 0.001.

OGD/R treatment resulted in obvious inflammation in rat spinal microglia. Blocking of Tim‐3 inhibited the expressions of TNF‐α, IL‐1β, IL‐6, and p‐p65 in OGD/R‐treated rat spinal microglia, partially abolishing OGD/R‐induced inflammation (Figure 7).

### 3.8 Tim‐3 Inhibition Attenuates Spinal Microglia Activation (M1 Polarization) Induced by OGD/R

**Figure 8:**
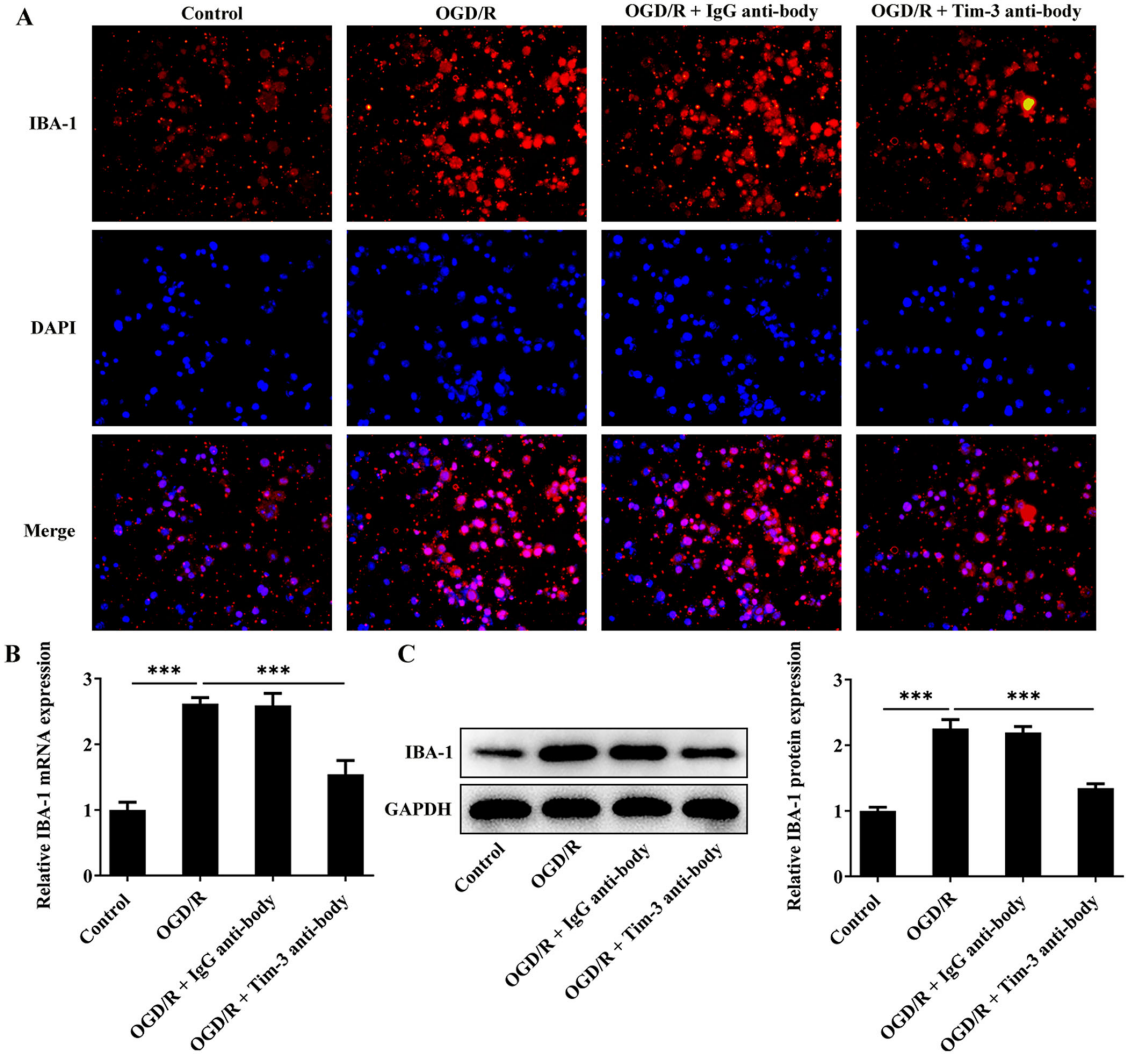
Tim‐3 inhibition attenuates spinal microglia activation induced by OGD/R. OGD/R‐treated rat spinal microglia was administrated with Tim‐3 antibody or lgG antibody. (A) IBA‐1 expression in rat spinal microglia was detected by immunofluorescence staining. (B, C) IBA‐1 mRNA and protein levels in rat spinal microglia were detected by RT‐qPCR and western blot assay, respectively. ***p < 0.001.

**Figure 9:**
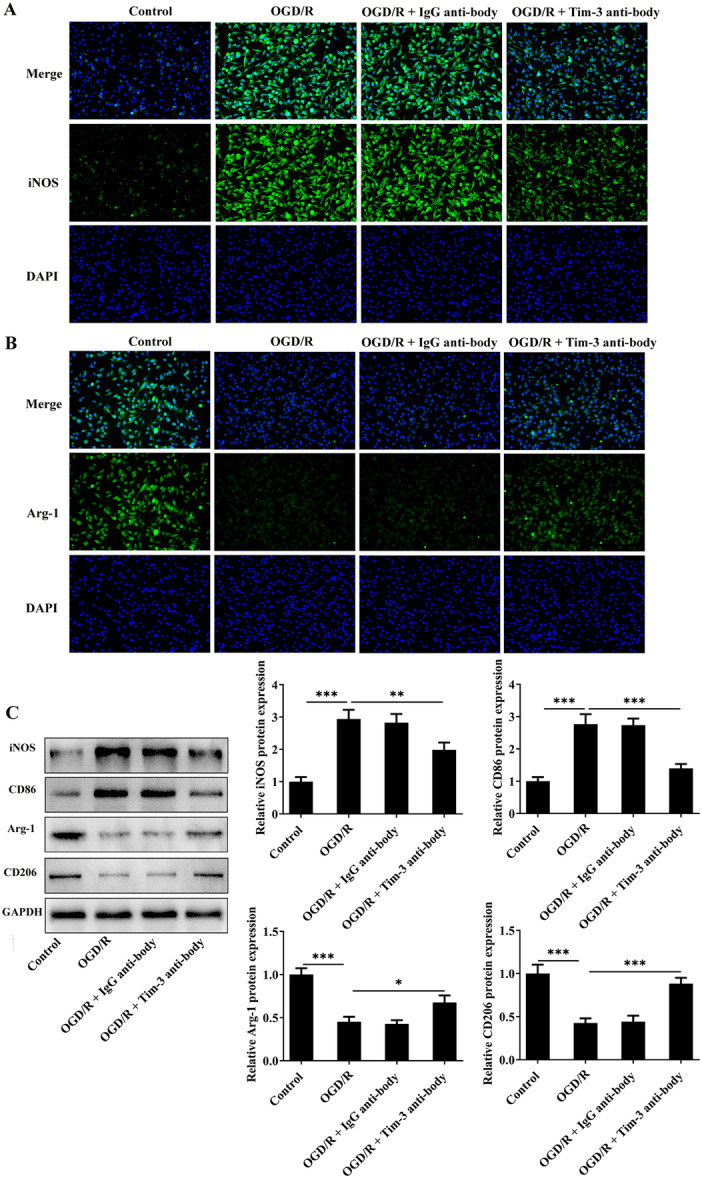
Tim‐3 inhibition attenuates M1 polarization of spinal microglia induced by OGD/R. OGD/R‐treated rat spinal microglia was administrated with Tim‐3 antibody or lgG antibody. (A) iNOS expression in rat spinal microglia was detected by immunofluorescence staining. (B) Arg‐1 expression in rat spinal microglia was detected by immunofluorescence staining. (C) Expressions of iNOS, CD86, Arg‐1, and CD206 in rat spinal microglia were detected by western blot assay. *p < 0.05, **p < 0.01, ***p < 0.001.

By immunofluorescence staining, RT‐qPCR and western blot assay, OGD/R treatment dramatically elevated IBA‐1 expression, inducing spinal microglia activation. Blocking of Tim‐3 partially restored the increased activation of spinal microglia, demonstrated by reduced IBA‐1 expression (Figure 8A–C). By immunofluorescence staining and western blot assay, OGD/R treatment markedly elevated expressions of iNOS and CD86 and notably reduced elevated expressions of Arg‐1 and CD206 in rat spinal microglia, indicating that OGD/R steered the polarization of spinal microglia towards the phenotype of M1. Furthermore, blocking of Tim‐3 promoted the polarization of spinal microglia from M1 to M2 phenotype, demonstrated by reduced expressions of iNOS and CD86 and elevated expressions of Arg‐1 and CD206 (Figure 9A–C).

## 4 Discussion

Various factors such as spinal fracture, spinal artery diseases, and spinal surgeries can lead to spinal cord ischemia, whereas restoration of blood flow is unable to improve spinal cord injury and even further aggravates the damage caused by primary ischemia [23, 24]. To this day, existing treatment strategies remains unsatisfactory. This current work was formulated to quest the biological role of Tim‐3 in SCIRI and probe into the intrinsic mechanisms.

Microglia activation is an essential pathological mechanism of SCIRI [25]. Neuroinflammatory response is closely related to the activation and polarization of microglia [26]. In the early stage after SCIRI, the microglia is activated, and then the activated microglia releases pro‐inflammatory chemokines to further aggravate SCIRI progression [27]. For instance, dexmedetomidine can suppress microglia activation through inactivating SNHG14/HMGB1 pathway to exert the therapeutic effects in SCIRI [21]. Delayed xenon post‐conditioning can improve neurological function in SCIRI by inhibiting microglia activation and reducing the release of pro‐inflammatory factors [25]. Curcumin can alleviate SCIRI by repressing microglia activation‐mediated neuroinflammation via inactivating Nrf2/NF‐κB axis [4]. This study adopted SCIRI rat model to examine the in vivo therapeutic potential of Tim‐3 antibody in SCIRI. It was verified that Tim‐3 was highly expressed in spinal cord tissues of SCIRI rats and blocking of Tim‐3 attenuated SCIRI‐induced pathological injury, neuronal apoptosis, neuroinflammation, and microglia activation (M1 polarization). Additionally, this study also adopted OGD/R‐treated rat spinal microglia to further identify the therapeutic role of Tim‐3 antibody in SCIRI in vitro. It was verified that Tim‐3 was highly expressed in OGD/R‐treated rat spinal microglia and blocking of Tim‐3 attenuated OGD/R‐induced inflammation and spinal microglia activation (M1 polarization).

To conclude, Tim‐3 antibody can exert therapeutic effects in SCIRI through inhibiting neuroinflammation and promoting microglia polarization from M1 to M2 phenotype. Still, application of transgenic animals is a good mean to further verify the therapeutic effects of Tim‐3 antibody in SCIRI. Moreover, analysis of the association between Tim‐3 expression and clinical features of SCIRI and rescue experiments for the intrinsic mechanisms should be performed in the future to support the obtained conclusion and excavate the predictive values of Tim‐3 antibody in SCIRI.

## Author Contributions

**Zhenxing Li:** Conceptualization; data curation; formal analysis; methodology; project administration; resources; writing–original draft; writing–review and editing. **Binbin Zhou:** Data curation; formal analysis; methodology; project administration; resources; writing–original draft; writing–review and editing. **Guanghui Chen:** Data curation; formal analysis; methodology; project administration; resources; writing–original draft. **Xiangyu Yang:** Data curation; formal analysis; methodology; project administration; resources; writing–original draft. **Han Su:** Data curation; formal analysis; methodology; project administration; software; writing–original draft. **Bolin Li:** Conceptualization; funding acquisition; investigation; validation; visualization; writing–original draft; writing–review and editing.

## Ethics Statement

The animal experimental protocol was approved by the Institutional Ethics Committee of First Affiliated Hospital of the Guangxi University of Chinese Medicine.

## Conflicts of Interest

The authors declare no conflicts of interest.

## Data Availability

The datasets generated or analyzed during this study are available from the corresponding author on reasonable request.
